# Thyroid hormone-induced cell death in sea urchin metamorphic development

**DOI:** 10.1242/jeb.244560

**Published:** 2022-12-01

**Authors:** Hannah Wynen, Elias Taylor, Andreas Heyland

**Affiliations:** University of Guelph, Integrative Biology, Guelph, ON, Canada, N1G 2W1

**Keywords:** Programmed cell death, *Strongylocentrotus purpuratus*, Apoptosis, Autophagy, Thyroxine, Endocrinology, Hormones

## Abstract

Thyroid hormones (THs) are important regulators of development, metabolism and homeostasis in metazoans. Specifically, they have been shown to regulate the metamorphic transitions of vertebrates and invertebrates alike. Indirectly developing sea urchin larvae accelerate the formation of juvenile structures in response to thyroxine (T4) treatment, while reducing their larval arm length. The mechanisms underlying larval arm reduction are unknown and we hypothesized that programmed cell death (PCD) is linked to this process. To test this hypothesis, we measured larval arm retraction in response to different THs (T4, T3, rT3, Tetrac) and assessed cell death in larvae using three different methods (TUNEL, YO-PRO-1 and caspase-3 activity) in the sea urchin *Strongylocentrotus purpuratus*. We also compared the extent of PCD in response to TH treatment before and after the invagination of the larval ectoderm, which marks the initiation of juvenile development in larval sea urchin species. We found that T4 treatment results in the strongest reduction of larval arms but detected a significant increase of PCD in response to T4, T3 and Tetrac in post-ingression but not pre-ingression larvae. As post-ingression larvae have initiated metamorphic development and therefore allocate resources to both larval and the juvenile structures, these results provide evidence that THs regulate larval development differentially via PCD. PCD in combination with cell proliferation likely has a key function in sea urchin development.

## Teaser

**Summary:** Thyroid hormones cause a significant increase in programmed cell death in post-ingression but not pre-ingression sea urchin larvae, indicating differential regulation of larval development by thyroid hormones via programmed cell death.

## INTRODUCTION

Programmed cell death (PCD) plays a critical role in homeostasis and development. PCD actively removes cells from tissues via coordinated and tightly regulated cellular and molecular mechanisms in response to internal cellular signals or external environmental cues (Elmore, 2007; Green and Fitzgerald, 2016). Apoptosis, arguably the best-known form of PCD, is a coordinated, energy-dependent process that involves a cascade of cellular events, including the activation of a highly conserved family of cysteine proteases called caspases, which lead to cell death (Elmore, 2007; Mocarski et al., 2012; Robertson et al., 2006; Ruocco et al., 2016; Sakamaki et al., 2014; Wynen and Heyland, 2021). Detailed information on PCD mechanisms originates from cell culture experiments and the majority of studies on whole organisms have been conducted in vertebrates (Bishop et al., 2006; Heyland and Moroz, 2006; Wynen and Heyland, 2021). The role of PCD in invertebrate development, specifically metamorphosis, remains poorly understood outside a select group of organisms, even though metamorphosis is widespread among animals and often involves apoptosis and other forms of PCD (Heyland and Moroz, 2006; Wynen and Heyland, 2021). For example, frogs and insects undergo drastic morphological and physiological transitions during metamorphic development and key events are regulated by hormones, such as tail resorption in frogs and removal of midgut tissue in insects (thyroid hormones and ecdysteroids, respectively). These processes are accompanied and in some cases even facilitated by PCD (Buszczak and Segraves, 2000; Ishizuya-Oka et al., 2010; Jiang et al., 1997; Nakajima et al., 2005; Romanelli et al., 2016; Tamura et al., 2015; Tata, 1993; Yaoita and Nakajima, 1997).

Metamorphic development (the formation of juvenile structures within the larval body) and settlement (the ecological transition from the larval into the juvenile habitat at the end of the larval period) are integral components of many marine invertebrate life histories (Bishop et al., 2006; Hadfield, 2000; Heyland and Moroz, 2006; Hodin, 2006; Pechenik et al., 1998). Despite the broad distribution of this developmental mode, little is known about mechanisms regulating larval and juvenile development. Research on echinoderms suggests that thyroid hormones (THs) can accelerate juvenile development while delaying larval development in some echinoid species such as sea urchins and sand dollars (Chino et al., 1994; Heyland and Hodin, 2004; Heyland and Moroz, 2006; Heyland et al., 2004). Furthermore, THs can be endogenously synthesized in the juvenile rudiment (Heyland et al., 2006a; Miller and Heyland, 2010, 2013) or derived from the unicellular algae on which the larvae feed (Van Bergeijk et al., 2013; Chino et al., 1994; Heyland and Hodin, 2004; Heyland et al., 2004). The development of the rudiment occurs after the ectoderm of the larva ingresses to contact the larval hydrocoel (Chino et al., 1994; Heyland and Hodin, 2014; Singh et al., 2018). This ingression is where the larva will form the juvenile rudiment and the completion of this step is known as the post-ingression stage, while anything prior to this ingression occurring is known as pre-ingression (Chino et al., 1994; Heyland and Hodin, 2014; Singh et al., 2018). This completion of ingression marks the beginning of metamorphic development and is therefore very useful for marking the different stages of larval development (Heyland and Hodin, 2014). The detailed mechanisms of TH action in sea urchins remain unknown. However, new evidence suggests that TH treatment affects the expression of skeletogenic genes and activates MAPK via a homologue of integrin αvβ3, a non-genomic TH transduction pathway that has been previously described in mammals (Taylor and Heyland, 2017, 2018, 2022; Brent, 2012; Davis et al., 2008; Lin et al., 2015; Martin et al., 2014; Yang et al., 2021).

Previous work has shown that PCD is involved in the metamorphic process and settlement among several marine invertebrate groups (Leise et al., 2004; Gifondorwa and Leise, 2006; Hadfield et al., 2015; Heyland et al., 2011b; Lutek et al., 2018). For example, apoptosis is responsible for the shedding of the velar lobes and apical organs in some indirectly developing veliger larvae of bivalves, scaphopods and gastropods (Heyland et al., 2011b; Kiss, 2010; Vogeler et al., 2021). Apoptosis in these organs is controlled by the neurotransmitter serotonin and inhibited by nitric oxide (NO) (Bishop and Brandhorst, 2001, 2003; Kiss, 2010). Inhibiting apoptosis with NO in molluscs inhibits metamorphosis completely in competent larvae, suggesting that apoptosis is critical for metamorphic development (Gifondorwa and Leise, 2006). In ascidians, apoptosis has been identified as a key player in the retraction of the larval tail during settlement (Karaiskou et al., 2015). Additionally, in the ascidian *Ciona intestinalis*, there has been a large expansion of the caspase family that may be partially explained by the role of apoptosis during tail regression (Weill et al., 2005). Indirectly developing sea urchins have been shown to use PCD during embryonic and larval development as well as the metamorphic transition (Lutek et al., 2018; Roccheri et al., 2002; Thurber and Epel, 2007; Voronina and Wessel, 2001). Apoptotic cells have been identified in the arms and ciliary bands of metamorphically competent larvae and juveniles (Agnello and Roccheri, 2010; Lutek et al., 2018; Roccheri et al., 2002; Thurber and Epel, 2007). Research on the purple sea urchin, *Strongylocentrotus purpuratus*, shows that histamine plays an important role, both aiding in the attainment of metamorphic competence and inhibiting settlement (Lutek et al., 2018). The inhibition of settlement seems to be partially due to histamine's role in inhibiting apoptosis in the larval arms (Lutek et al., 2018). Not only is settlement inhibited when apoptosis is inhibited but also inhibiting apoptosis in the arm tips causes cells to be expelled from those areas in a process mediated by histamine receptor action (Lutek et al., 2018).

Previous work on sea urchins shows that THs affect both larval and juvenile skeletal structures (Armstrong and Lessios, 2015; Chino et al., 1994; Heyland et al., 2011a; Heyland and Hodin, 2004; Heyland et al., 2006a, 2004; Lutek et al., 2018; Taylor and Heyland, 2018). Specifically, TH treatment reduces larval arm length in some echinoid species (Armstrong and Lessios, 2015; Chino et al., 1994; Heyland and Hodin, 2004; Heyland et al., 2006a, 2004) while accelerating skeleton formation in embryos (Taylor and Heyland, 2018). However, it remains unknown whether this arm length reduction in larvae is a result of PCD, whether it is regulated by TH action, and whether this effect is stage dependent. Here, we tested the hypothesis that larval arm length reduction is a result of PCD in larvae of *S. purpuratus* and that this process is regulated by THs. To assess the specificity of THs, we tested several different forms of the hormone (T4, T3, Tetrac and rT3) on PCD using three different but complementary markers (caspase-3, TUNEL and YO-PRO-1). We also compared the impact of these hormones pre- and post-ingression of the larval ectoderm. This development stage is a critical point in larval development as it demarcates the beginning of juvenile development (Bessodes et al., 2012; Byrne et al., 2018; Heyland and Hodin, 2014).

## MATERIALS AND METHODS

### Fertilization and larval culture maintenance

Adult purple sea urchins, *Strongylocentrotus purpuratus* (Stimpson 1857), were shipped to the University of Guelph from Monterey, CA, USA, where they were collected by diving. Once at the Hagen Aqualab (University of Guelph) they were divided by sex and kept in tanks of artificial seawater (created by adding 32 g l^−1^ Instant Ocean 5082654, and 0.05 g l^−1^ pH 8.3 Reef Buffer) that had a temperature of approximately 12–14°C, salinity of 32 ppt and pH of 8.0–8.3. Adults were fed kelp (*Macrocystis pyrifera* and/or *Kombu* spp*.*) twice a week and the tanks were cleaned 3 times a week.

Gametes were collected by injecting 0.5–1.5 ml of 0.5 mol l^−1^ KCl (depending on the size of the animal) into the gonad of adult purple sea urchins to induce spawning. Sperm was collected in a dry pipette before being mixed into 5 ml of filtered artificial seawater (FASW). Females were inverted over a beaker of FASW to collect their eggs. Once the eggs settled at the bottom of the beaker, they were washed twice with FASW. Diluted sperm (1 ml) was slowly mixed into the beaker until more than 85% of the eggs had fertilization envelopes, which indicated successful fertilization. The eggs were washed once more and 24 h later, hatched embryos were transferred to 2 l beakers of FASW with a salinity of 32 ppt. The larvae were kept on a 12 h:12 h light:dark cycle. The larvae were fed a combination of 2000 cells ml^−1^ *Dunaliella tertoliecta* and 4000 cells ml^−1^ *Rhodomonas salina* algae 3 times per week. Cleaning and water changes were also performed 3 times per week. Larvae were collected for experiments at 10–11 days (pre-ingression) and 20–22 days (post-ingression) and the synchrony of developmental stages was confirmed via microscopy (Heyland and Hodin, 2014).

### TH preparation and treatment

The THs reverse-triiodothyronine (rT3), triiodothyronine (T3), thyroxine (T4) and tetraiodothyroacetic acid (Tetrac) were dissolved at 100 mmol l^−1^ in DMSO (THs from Sigma-Aldrich, product numbers T-075, T0281, IRMM468, T3787). They were then diluted in FASW to a 1 mmol l^−1^ concentration before being stored at −20°C. Prior to experiments, these hormones were diluted in FASW to their required concentrations.

For the morphology experiments, larvae were exposed for 24 h to seawater alone (control) or containing rT3 at a concentration of 10^−7^ mol l^−1^, T3 at 10^−7^ or 10^−9^ mol l^−1^, T4 at 10^−7^ or 10^−9^ mol l^−1^, and Tetrac at 10^−7^ or 10^−9^ mol l^−1^. For all apoptosis staining experiments, larvae were exposed for 24 h to seawater alone (control) or containing 10^−7^ mol l^−1^ rT3, 10^−7^ mol l^−1^ T3, 10^−7^ mol l^−1^ T4 or 10^−9^ mol l^−1^ Tetrac.

### Detecting early-stage apoptosis using YO-PRO-1

YO-PRO-1 (YP1) is a nuclear stain that detects cell death by binding to the DNA. As apoptosis causes membrane instability, YP1 can enter dying cells but is too large to pass through the membrane of living cells. YP1 is a sensitive early marker of apoptosis (Fujisawa et al., 2014). In order to distinguish apoptotic from necrotic cells, propidium iodide (PI) was used alongside YP1. PI is a cationic fluorochrome which is able to enter cells with severely compromised plasma membranes (Zhao et al., 2010). We also used Hoechst 33342 stain to identify nuclei of all cells.

PI, YP1 and Hoechst 33342 were used as described below. Larvae were incubated overnight at 12°C in 6-well plates containing 10 ml of one of the five treatments and approximately 100 larvae per well. An additional heat shock group was included to verify whether the stain showed a significant difference compared with the seawater control group. Larvae in the heat shock group were incubated at 28°C for 2 h.

The protocol used for these stains is a modified version of the Invitrogen Membrane Permeability/Dead Cell Apoptosis Kit with YO-PRO^®^-1 and PI for Flow Cytometry (Invitrogen V13243). Hoechst 33342 (Invitrogen H3570; hereafter Hoechst) stock was prepared by dissolving 100 mg of the desiccated stock in 10 ml of deionized water to create a 10 mg ml^−1^ stock. PI (Invitrogen Y3603) stock was prepared by dissolving 1 mg of desiccated stock in 1 ml of deionized water to create a 1 mg ml^−1^ stock. YP1 (Invitrogen Y3603) arrived diluted as a 100 µmol l^−1^ solution in DMSO. Immediately prior to the experiment, YP1 working stock was diluted 1:1 in seawater in order to make dissolution in the well plates easier. For this reason, PI working stock was also diluted 1:1 in seawater and Hoechst was diluted 1:5 in seawater immediately before use. After 24 h incubation of larvae in seawater alone or TH treatment, 1 μl of PI dilution and 1 μl of YP1 dilution was added to each 10 ml well. Larvae were then incubated for 15 min at 12°C. After 15 min, 1 μl of diluted Hoechst was added to each 10 ml well. Larvae were then incubated for an additional 15 min. After this incubation, 1020 larvae were mounted on a coverslide and imaged within a 30 min period as the stains are time sensitive. This process was repeated until there was a minimum of 10, ×200 images for each treatment group with well-defined staining. As this was a live stain and any movement by the larval arms could affect image clarity, ×600 images were not collected for this experiment. Exposure time was determined by finding an apoptotic cell on a larval arm from the seawater control group and adjusting the exposure so that it was still visible with minimal background fluorescence. Exposure time was kept consistent for all acquisitions. The stains YP1, PI and Hoechst have excitation/emission wavelengths of 491/509 nm, 535/617 nm and 350/461 nm, respectively. Larvae were imaged at ×300 magnification using the Nikon fluorescence filter cubes for DAPI (Hoechst), GFP/FITC (YP1) and TRITC/CY3 (PI), respectively (www.microscope.healthcare.nikon.com).

### Detecting late-stage apoptosis using TUNEL

Terminal deoxynucleotidyl transferase (TdT) dUTP Nick-End Labelling (TUNEL) is a commonly used apoptosis stain (Kyrylkova et al., 2012). TUNEL adds labelled dUTPs to the 3′ hydroxyl termini of DNA ends, causing DNA in apoptotic cells to fluoresce.

A commercial kit (Click-iT™ Plus TUNEL Assay for In Situ Apoptosis Detection, Alexa Fluor™ 488 dye kit Invitrogen C10617) was used to identify cells in the later stages of apoptosis. Larvae were incubated overnight in 6-well plates containing 10 ml of one of the five treatments and approximately 100 larvae per well. An additional seawater alone group was included that was later treated with DNAse (as suggested by the manufacturer) as a positive control. Larvae were incubated for 24 h at 12°C, after which they were collected in 1.5 ml Eppendorf tubes and excess water was removed until 500 μl remained. Larvae were fixed by adding 160 μl of 16% paraformaldehyde (4% final paraformaldehyde concentration in seawater) and incubated overnight at 4°C. Larvae were then washed in 500 μl of phosphate-buffered saline (PBS). Post-ingression larvae were washed 5 times whereas pre-ingression larvae were only washed 3 times. This was both to minimize lost larvae during the wash process and to prevent damage to the larvae as the pre-ingression larvae tend to be more fragile than the post-ingression larvae. The original Click-iT™ Plus TUNEL kit protocol was optimized for fixed tissue samples on slides; however, because of the delicate nature of the larvae, staining was conducted in 1.5 ml Eppendorf tubes. The diluted Hoechst stain (100 µl) was added to each sample. Samples were then incubated for 20 min at room temperature, protected from light. After this incubation, Hoechst solution was removed and samples were rinsed twice in 1× PBS. Finally, samples were stored in 500 µl of 1,4-diazabicyclo[2.2.2]octane (DABCO) at 4°C protected from light. The DABCO solution was created by adding 0.1 g of DABCO to a 15 ml Falcon tube with 1 ml of 1 mol l^−1^ Tris HCl pH 8.5; the solution was mixed until all visible DABCO crystals were dissolved, then 9 ml of 100% glycerol, heated in a microwave for 10 s, was added to the Falcon tube. Finally, the solution was gently mixed in order to prevent the creation of bubbles and cooled at room temperature before storing at 4°C.

### Detecting caspase-3 activity through immunohistochemistry

Caspase-3 Rabbit IgG polyclonal antibody (Invitrogen MA1-91637) was used for immunohistochemical detection of caspase-3 activity in larval tissues. The specific caspase-3 antibody was chosen because it has been used successfully in previous experiments with sea urchin embryos (Wei et al., 2012). Larvae were fixed in 4% paraformaldehyde as detailed above for TUNEL fixation, and incubated according to their age: pre-ingression larvae were incubated for 30 min and post-ingression larvae were incubated for 1 h. Larvae were then rinsed twice with 100% ice-cold methanol to remove the paraformaldehyde before being stored at −20°C.

Larvae were rinsed in 50 μl of 0.3% PBS with 0.1% Tween-20 (PBST) for 10 min. This was repeated 3 times for pre-ingression larvae and 5 times for post-ingression larvae. Blocking solution (40 ml) was prepared using 80 mg of bovine serum albumin (BSA), 400 μl of goat serum and 0.3% PBST as a base. Larvae were rinsed in the blocking solution for 1 min and then incubated in 100 μl of blocking solution at 4°C overnight. Primary antibody solution was diluted to 1 μg ml^−1^ and 1 μl was then diluted in 125 μl of blocking solution (0.008 µg ml^−1^) and added to each tube before incubation overnight at 4°C. Larvae were then rinsed in 0.3% PBST, 5 times for 6 min. The secondary antibody solution was prepared by adding 20 μl of 8 mg ml^−1^ goat anti-rabbit FITC-conjugated antibody (Invitrogen) to 9.98 ml of 0.3% PBST to a final concentration of 0.016 mg ml^−1^; 250 μl was added to each tube followed by incubation at room temperature for 4 h hours protected from light. Larvae were rinsed 1–2 times in 0.3% PBST for 5 min protected from light. Finally, larvae were rinsed in 1× PBS and stored in 500 μl of DABCO. Hoechst stock was prepared as above by dissolving 100 mg of the desiccated stock in 10 ml of deionized water to create a 10 mg ml^−1^ working stock. Prior to imaging, 1 μl of Hoechst working solution was diluted in 2 ml of DABCO for a final concentration of 0.005 mg ml^−1^; 50 μl of this solution was then added to the bottom of each Eppendorf tube and incubated for 20 min protected from light. Hoechst has an excitation/emission wavelength of 350/461 nm. The FITC fluorophore used has an excitation/emission wavelength of 491/516 nm. Larvae were imaged at ×60 magnification using Nikon Longpass fluorescence filter cubes for DAPI (Hoechst), GFP/FITC (YP1) and TRITC/CY3 (PI), respectively (www.microscope.healthcare.nikon.com).

### Image deconvolution and cell counting

Prior to analysis, images were batch deconvolved using NIS-Elements AR analysis module software. 3D deconvolution was performed using widefield modality. Images were then examined using Fiji software (imagej.net). First, a section of the post-oral arm was established as a region of interest (ROI). The ROI was selected and isolated from the rest of the image before it was measured automatically.

For YP1 staining, cell types were categorized and recorded. Stained ciliated, skeletal and epithelial cells were initially identified based on their morphology and location. These cells were then further distinguished based on their staining. Apoptotic ciliated cells were identified as small, circular fluorescent cells on the outer layers of the arm that were only stained by Hoechst and YP1 (Fig. 2A). Apoptotic epithelial cells were defined as large cells found along the outer layers or tip of the arm that were only stained by Hoechst and YP1 (Fig. 2C). Apoptotic skeletal cells are the same size as epithelial cells but are found along the skeletal rod and were only stained by Hoechst and YP1 (Fig. 2B). Finally, fluorescent cells in the FITC channel were counted for each category. Any cells that were stained by both YP1 and PI were considered necrotic and were excluded from counts (Fig. 2A′,B′,C′). The number of cells in each category as well as the total number of cells was then divided by the area of the ROI in order to calculate the apoptotic cell density (Fig. 4).

For TUNEL staining, contrast and brightness among images were adjusted by identifying one clear apoptotic cell in the control treatment and adjusting the brightness and contrast so that it was clearly visible while background fluorescence was minimal (Fig. 4). These values were then used for the rest of the images taken from that date. This process was repeated for each of the three imaging groups. After this, any visible fluorescent cells were counted as apoptotic. The number of apoptotic cells was then divided by the area of the ROI in order to calculate the average apoptotic cell density (Fig. 5). The same approach was taken to identify and quantify caspase-3 immunohistochemistry positive cells (Figs 6 and 7).

### Statistical analysis

For the YP1 experiment, there were four dependent variables used as markers of apoptotic cell density: apoptotic cell density, apoptotic ciliated cell density, apoptotic skeletal cell density and apoptotic epithelial cell density. These variables made it possible to identify whether certain THs increased apoptotic cell density overall and whether they increased it in a tissue-specific manner. For the TUNEL experiment, there were two dependent variables: number of apoptotic cells per arm and apoptotic cell density. These variables made it possible to identify whether there was an overall increase in the number of apoptotic cells or whether the number was increased relative to the area of the arm. For the immunohistochemistry experiments, the dependent variables were: caspase-3 positive cell density, the number of positive signals per cell and caspase-3 signal density. The caspase-3 positive cell density and the number of positive signals per cell were measured because it is important to identify whether THs increase the quantity of cells expressing active caspase-3. While this is important information, it can be difficult to accurately attribute a positive signal to the correct cell based on the signal location and the clarity of the image and so by also measuring the caspase-3 signal density, it is possible to accurately determine whether THs increase the overall amount of active caspase-3. However, this provides no information as to whether they increase it more in some cells or whether the activation is consistent.

ANOVA or MANOVA commands in SPSS v25 were used to analyse the overall effect of factors on dependent variables and *post hoc* comparisons using Tukey's HSD commands. Factors tested were hormone treatment, developmental stage and arm type, depending on the analysis. Normality of all data was checked using *P–P* and *Q–Q* plots prior to running MANOVA commands in SPSS v25. Statistically significant differences between hormone treatments, developmental stages or arm types are not indicated directly in figures but are provided in Table S1 and summarized in Table 1. We also provide average differences from comparisons in the text as *X*_d_=[difference to control]; *P*=[significance].

**Table 1.**
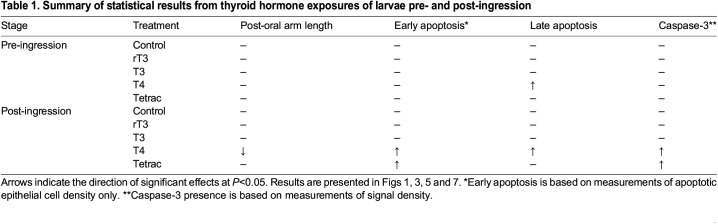
Summary of statistical results from thyroid hormone exposures of larvae pre- and post-ingression

## RESULTS

We conducted a series of experiments to assess the impact of TH treatment on arm length and markers of cell death. Specifically, we measured the arm length changes in response to T4, rT3, T3 and Tetrac. We also conducted several control experiments using heat-shock and DNAse treatment of larvae to induce apoptosis. We measured both early- (YP1) and late-stage (TUNEL) apoptosis as well as activated caspase-3 levels in larvae of the sea urchin *S. purpuratus*.

### T4 and Tetrac treatment result in arm length reduction of post-ingression larvae

**Fig. 1.**
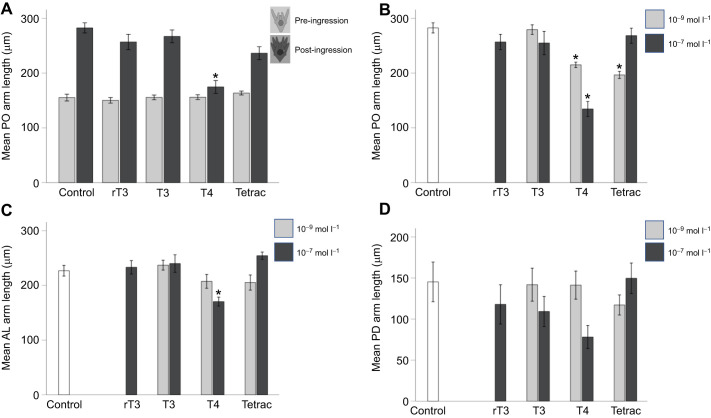
**Effect of thyroid hormone (TH) treatment on arm length in pre- and post-ingression sea urchin larvae.** Larvae were treated with seawater alone (control), reverse-triiodothyronine (rT3; TH control), triiodothyronine (T3), thyroxine (T4) or tetraiodothyroacetic acid (Tetrac). (A) T4 (thyroxine) and Tetrac reduce post-oral (PO) arm length in post-ingression larvae but not pre-ingression larvae. (B–D) In post-ingression larvae, post-oral (PO; B), anterolateral (AL; C) and post-dorsal (PD; D) arms are reduced in response to T4 and Tetrac treatment; however, Tetrac consistently shows an inverse dose–response relationship with respect to arm length. Data are means±1 s.e.m. *Significance of *P*<0.05.

We measured the length of post-oral (PO), post-dorsal (PD) and anterolateral (AL) arm length in response to two concentrations (10^−7^ and 10^−9^ mol l^−1^) of T4, rT3, T3 and Tetrac, and in pre- and post-ingression larvae (Fig. 1). We found that T4 treatment led to a significant reduction in arm length. We also found that Tetrac treatment resulted in arm length reduction at the low but not the high concentration.

We conducted a three-way ANOVA to test whether TH treatment resulted in arm retraction differences in pre- versus post-ingression larvae. Although we identified a significant effect of treatment (*F*_4,287_=16.071, *P*<0.001) and arm type (*F*_2,287_=194.806, *P*<0.001), we did not identify a main effect between pre- and post-ingression larvae (*F*_1,287_=3.528, *P*=0.061; Fig. 1A). We then analysed each arm type separately, using a two-way ANOVA (treatment and developmental stage). Main effects and interactions were statistically significant for PO arms (treatment: *F*_4,121_=20.850, *P*<0.001; stage: *F*_1,121_=140.387, *P*<0.001; treatment×stage: *F*_3,121_=26.284, *P*<0.001) and AL arms (treatment: *F*_4,121_=7.167, *P*<0.001; stage: *F*_1,121_=108.953, *P*<0.001; treatment×stage: *F*_3,121_=12.621, *P*<0.001). We did not include PD arms in this analysis as PD arms develop after ingression.

In order to compare the effect of hormone treatment on arm length, pre- and post-ingression, we conducted *post hoc* comparisons using Tukey's HSD correction for each arm type (Fig. 1B–D). Pre-ingression, no specific hormone treatment resulted in a significant reduction of arm length for PO and AL arms in the *post hoc* comparisons (see Table S1 for details), although treatment had a significant overall effect on AL arm length pre-ingression (*F*_3,76_=2.973, *P*=0.037). Post-ingression, both PO and AL arm length were significantly affected by hormone treatment (PO: *F*_4,45_=15.978, *P*<0.001; AL: *F*_4,45_=8.385, *P*<0.001) but PD arm length was not (*F*_4,45_=2.080, *P*=0.099). *Post hoc* comparison revealed significant differences in PO arm length between the control and T4 treatment (*X*_d_=148.103; *P*<0.001), the rT3 and T4 treatment (*X*_d_=122.509; *P*<0.001), the T3 and T4 treatment (*X*_d_=120.529; *P*<0.001) and the Tetrac and T4 treatment (*X*_d_=133.904; *P*<0.001). We noticed an inverse dose dependence for all arm types in the Tetrac treatment, resulting in a larger reduction in arm size in the 10^−9^ mol l^−1^ treatment compared with the 10.7 mol l^−1^ treatment. We tested these results by comparing the treatments with two doses (Tetrac, T3 and T4) separately. We found that for PO arms at 10^−7^ mol l^−1^ concentration, T4 resulted in reduced arm length, compared with T3 (*X*_d_=130.671; *P*<0.001) and Tetrac (*X*_d_=136.855; *P*<0.001). For 10^−9^ mol l^−1^ concentration, both T4 (*X*_d_=64.247; *P*<0.001) and Tetrac (*X*_d_=82.663; *P*<0.001) resulted in reduced PO arm length in comparison to T3. Note that this inverse dose dependence was not significant for either AL or PD arms.

### T4 and Tetrac treatment result in increased levels of the early apoptosis marker YP1 in post-ingression larvae

**Fig. 2.**
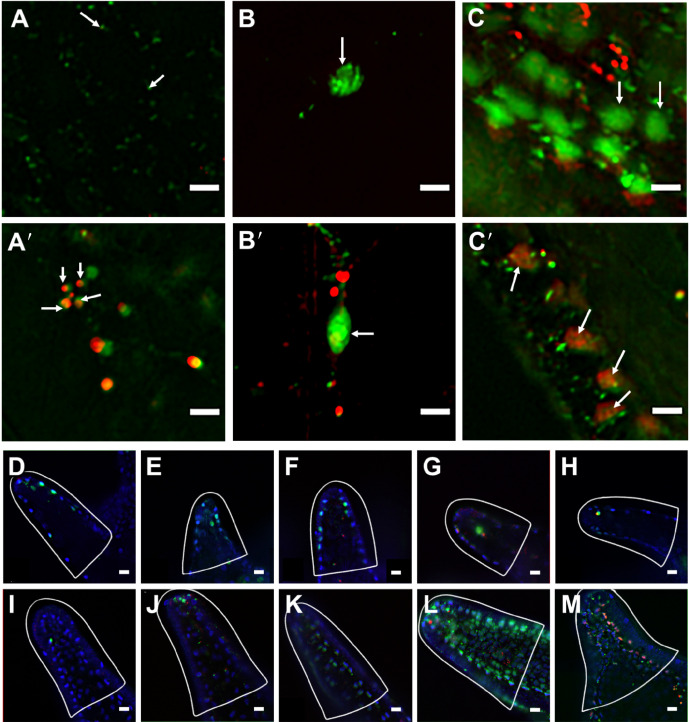
**T4 treatment results in increased levels of early apoptosis measured by YO-PRO-1 and propidium iodide in post-ingression sea urchin larval arms.** (A–C and A′–C′) Representative images from post-ingression larvae (20–22 days post-fertilization, dpf), exposed to one of five hormone treatments (control, rT3, T3, T4 or Tetrac; see Materials and Methods for details), featuring the cell categories (ciliated, mesenchymal, epithelial) quantified in this study using YO-PRO and propidium iodide (PI) staining, imaged under ×300 magnification (scale bars: 5 µm). (A) Apoptotic ciliated cells: arrows point to two small green cells with no overlapping red signal (Tetrac treatment). (A′) Apoptotic ciliated cells with necrosis: arrows indicate a cluster of four apoptotic bodies. It is difficult to distinguish between early apoptotic ciliated cells, necrotic ciliated cells and apoptotic bodies; however, apoptotic bodies and necrotic ciliated cells fluoresce when stained with PI. Additionally, apoptotic bodies are often found in tightly packed clusters (Tetrac treatment). (B) An apoptotic mesenchymal cell associated with skeletal rods (arrow) with no overlapping PI signal (control treatment). (B′) An apoptotic mesenchymal cell associated with a skeletal cell (arrow) that is not in the early stages of apoptosis as the cell shows a positive PI signal, indicating that it is either necrotic or past the early stages of apoptosis. Several necrotic cells can also be seen in the background. (C) Apoptotic epithelial cells (arrows): larger tightly packed cells with no overlapping red signal, with some necrotic cells in background (T3 treatment). (C′) Five epithelial cells with PI staining. These cells are either dead or in the very last stages of apoptosis, because they feature a clear PI signal and apoptotic bodies (arrows). (D–M) Representative images of early apoptosis in response to different TH treatments (D,I: control; E,J: rT3; F,K: T3; G,L: T4; H,M: Tetrac) for 24 h before staining with YO-PRO-1, PI and Hoechst pre-ingression (D–H; 10–11 dpf) and post-ingression (I–M; 20–22 dpf) and imaged at ×100 magnification (scale bars: 10 µm). Arms are outlined with white lines. The strongest YO-PRO-1 signal was detected in the T4 treatment post-ingression, and the strongest PI signal was detected in Tetrac treatment post-ingression. Cells stained with YO-PRO-1 are green, cells stained with PI are red, cells stained with Hoechst are blue.

We measured YP1 intensity in PO arms and identified three separate cell types for the analysis: ciliated, skeletal and epithelial, as well as all early apoptosis cells. We conducted these measurements on PO arms as they showed the strongest arm retraction response to T4 treatment (Fig. 1A). Fig. 2 shows representative images of YP1 staining, illustrating the cell types as well as the criteria for positive YP1 staining (Fig. 2A–C) and representative images of hormone-treated larvae (Fig. 2D–M). As outlined in the Materials and Methods, YP1 stain was applied in combination with PI. If a cell takes up both YP1 and PI it will fluoresce in both green and red and this indicates that the cell is experiencing dissolution of the nucleus and is either necrotic or past the point of early apoptosis (Fig. 2B′). Cells that were positive for both YP1 and PI were excluded from the count. Identification of cell type was conducted by identifying common morphological traits of each cell type and classifying cells based on those traits. As it can be difficult to distinguish these morphological characteristics in differential interference contrast (DIC) microscopy, only cells that were YP1 positive could be classified. Apoptotic ciliated cells were classified as small round cells that were typically on the outside layers or sides of the larval arm and were only positive for YP1 (Fig. 2A). These were separated from necrotic ciliated cells and apoptotic bodies, which were positive for both YP1 and PI (Fig. 2A′). Additionally, while they were very similar in size and shape, it was possible to distinguish apoptotic ciliated cells from apoptotic bodies. Typically, apoptotic ciliated cells were somewhat spaced out and often appeared in very loose linear patterns (Fig. 2A). Apoptotic bodies, in contrast, were tightly clustered in almost circular patterns (Fig. 2A′). Skeletal cells were identified primarily based on their location (Fig. 2B). Apoptotic skeletal cells rested along the skeletal rods within the larval arms, were often isolated from other apoptotic cells and were positive for PI. Once again, these cells were distinguished from necrotic skeletal cells by their lack of PI staining, which indicates an intact nucleus (Fig. 2B′). It can be difficult to identify skeletal cells because of their similarity in shape and location to primary mesenchyme cells (PMCs). Finally, apoptotic epithelial cells were classified based on their large size, proximity to other cells, location on the outer layers of the larval arms and YP1 staining (Fig. 2C). Cells that met these criteria but were also positive for PI were considered necrotic and were not counted (Fig. 2C′).

**Fig. 3.**
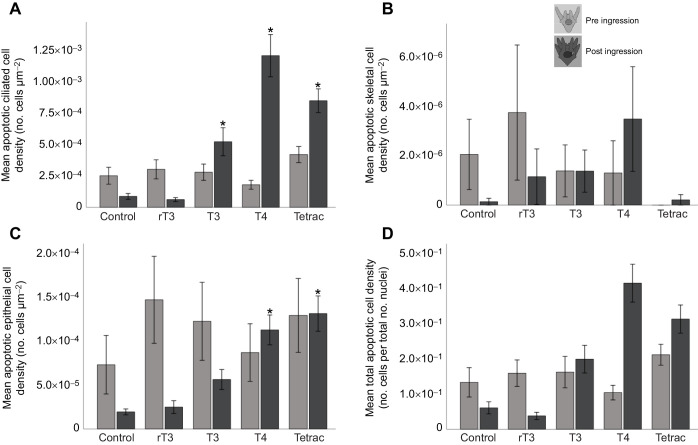
**T3, T4 and Tetrac result in increased levels of early apoptosis measured by YO-PRO-1 and PI in ciliated and epithelial cells in larval arms of post-ingression but not pre-ingression larvae.** Cells stained with YO-PRO-1 and PI were acquired and processed for intensity quantification and cell count. Cell density for apoptotic ciliated (A), skeletal (B) and epithelial (C) cells was calculated by subtracting the necrotic signal (PI) from the apoptotic signal (YO-PRO-1) in these cell types and dividing by the surface area. In order to validate this approach, apoptotic cells were also counted manually and divided by the manual count of all nuclei within the area (D). Larvae were treated for 24 h in all experiments: control, rT3, T3, T4 and Tetrac. Data are means±1 s.e.m. *Significance of *P*<0.05.

We conducted a two-way MANOVA with hormone treatment and developmental stage as factors and cell type as the dependent variable. We found that early apoptosis in ciliated cells (Fig. 3A) was affected by both treatment (*F*_4,175_=16.999, *P*<0.001) and developmental stage (*F*_1,175_=23.342, *P*<0.001). We also found that the effect of treatment was different between pre- and post-ingression larvae, expressed by a statistically significant interaction between treatment and developmental stage (*F*_4,175_=18.961, *P*<0.001). Early apoptosis in skeletal cells (Fig. 3B) as measured by cell density was impacted by neither hormone treatment (*F*_4,175_=0.000, *P*=1.000) nor developmental stage (*F*_1,175_=0.000, *P*=1.000) and no significant interaction between treatment and developmental stage was detected (*F*_4,175_=0.000, *P*=1.000). Apoptotic epithelial cell density (Fig. 3C) was significantly impacted by developmental stage (*F*_1,175_=4.831, *P*=0.029), but not treatment (*F*_4,175_=1.870, *P*=0.118), and no significant interaction was detected (*F*_4,175_=1.860, *P*=0.120). Total apoptotic cell density (Fig. 3D), while not impacted by treatment (*F*_4,175_=1.543, *P*=0.192), changed in response to developmental stage (*F*_4,175_=56.461, *P*<0.001) and showed a significant interaction between treatment and developmental stage (*F*_4,175_=4.728, *P*=0.001).

In order to assess the impacts of treatment on early-stage apoptosis, we then separately analysed pre- and post-ingression stages using *post hoc* comparisons with Tukey's HSD correction. Pre-ingression, treatment did not result in any change of early apoptosis in ciliated (*F*_4,175_=2.143, *P*=0.082), skeletal (*F*_4,175_=0.831, *P*=0.509) or epithelial cells (*F*_4,175_=0.524, *P*=0.719). We did find that hormone treatment had a significant impact on early apoptosis in pre-ingression larvae when measured by the total number of apoptotic cells (*F*_4,175_=2.810, *P*=0.030). *Post hoc* comparisons between TH treatments and the control did not reveal any changes in overall early apoptosis cell density (Table S1).

For post-ingression larvae, TH treatment did result in a significant change of early apoptosis in ciliated cell density (*F*_4,175_=24.517, *P*<0.001; Fig. 3A), epithelial cell density (*F*_4,175_=14.646, *P*<0.001; Fig. 3C) and total early apoptosis cell density (*F*_4,175_=3.522, *P*=0.010; Fig. 3D). No significant impact of treatment was detected for skeletal cell density (*F*_4,175_=1.293, *P*=0.279; Fig. 3B). *Post hoc* comparisons between TH treatments and the control revealed a significant increase of early apoptosis in ciliated cells for T3 (*X*_d_=−4.347E−004; *P*=0.039), T4 (*X*_d_=−1.1E−003; *P*<0.001) and Tetrac (*X*_d_=−7.610E−004; *P*<0.001) but not rT3 (*X*_d_=2.516E−005; *P*=1.000). We found an increase in early apoptosis in epithelial cell density with T4 (*X*_d_=−9.313E−005; *P*<0.001) and Tetrac (*X*_d_=−1.117E−004; *P*=0.039) treatment but not rT3 (*X*_d_=−5.474E–006; *P*=0.998) or T3 (*X*_d_=−3.679E−005; *P*=0.344) treatment. For total early apoptosis cell density, we did not find any significant difference between the control treatment and any of the TH treatments (Table S1).

### T4 results in increased levels of the late-stage apoptosis marker TUNEL in post-ingression larvae

**Fig. 4.**
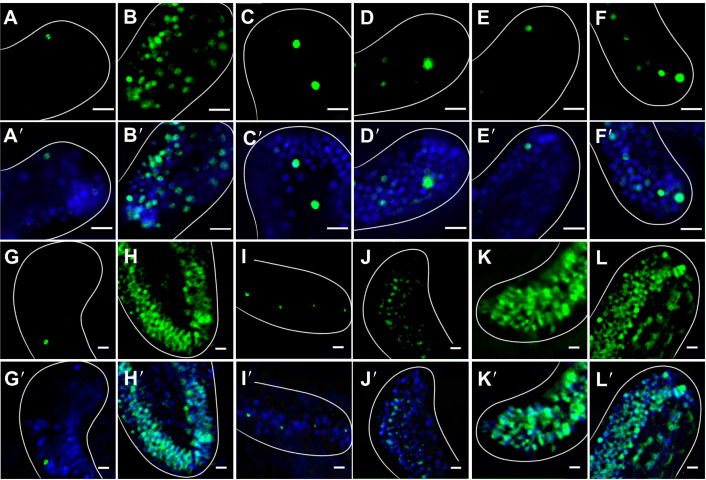
**T4 and Tetrac treatment result in the largest number of apoptotic cells (late-stage cell death) in the PO arms of sea urchin larvae, as detected by TUNEL staining.** Representative images of TUNEL (A–L) and TUNEL plus Hoechst (A′–L′) staining in response to TH treatment [A,A′,G,G′: control; B,B′,H,H′: positive control (heat-shock treatment); C,C′,I,I′: rT3; D,D′,J,J′: T3; E,E′,K,K′: T4; F,F′,L,L′: Tetrac] in the larval arms of pre-ingression (A,A′–F,F′; 10–11 dpf) and post-ingression (G,G′–L,L′; 20–22 dpf) larvae. Cells stained with TUNEL are green and cells stained with Hoechst are blue. Scale bars in all images: 10 μm. Larval arms are outlined with white lines. For quantitative results, see Fig. 5.

In order to assess the impacts of TH treatment on late-stage apoptosis, we measured TUNEL-positive (TUNEL+) cells in PO and PD arms of pre- and post-ingression larvae. Fig. 4 shows representative examples of TUNEL+ cells in PO larvae arms. TUNEL+ cells were identified throughout the larval arms. Because of the fixation process used, the cells experienced some bloating, which made it impossible to correctly distinguish the different cell types. Cells that were TUNEL+ fluoresced green when excited with FITC (Fig. 4). Hoechst was used as a counterstain because of its ability to stain all DNA, which allowed us to check that every TUNEL+ cell overlapped with a Hoechst-positive cell and that cells were not counted multiple times in *z*-projections. We conducted a positive control using heat-shock treatment to ensure that TUNEL detected induced late-stage cell death in our experiments and found a consistent increase of TUNEL+ cells in the positive controls (Table S1).

**Fig. 5.**
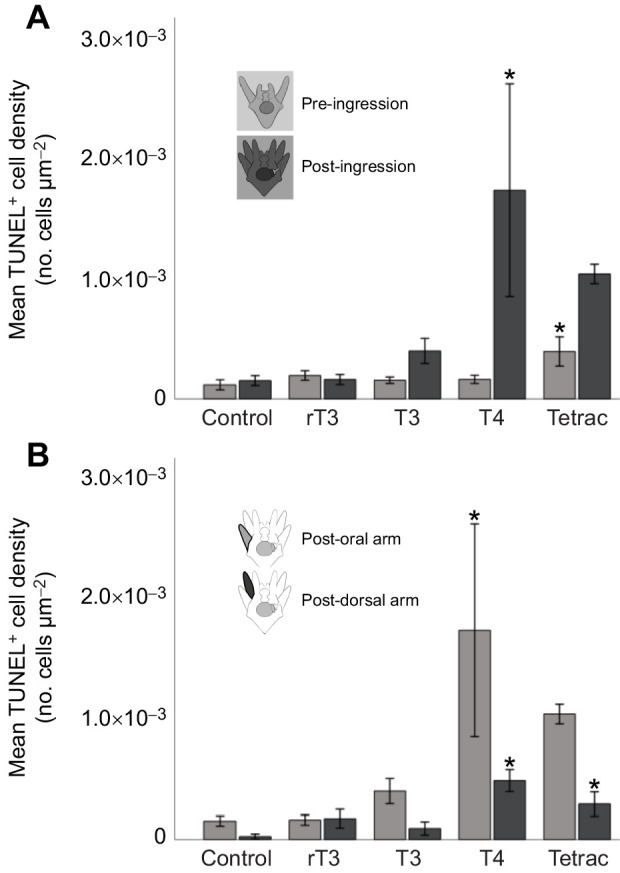
**T4 and Tetrac treatment result in increased levels of apoptosis (late-stage cell death) in post-ingression compared with pre-ingression larvae, and in PO arms compared with PD arms, post-ingression, as detected by TUNEL staining.** (A) Apoptotic cell density increased in larvae treated with Tetrac in pre-ingression larvae, and T4 treatment resulted in increased apoptosis levels post-ingression. (B) Post-ingression, we found higher levels of apoptosis measured by TUNEL in PO arms compared with PD arms in response to T4 treatment. We also found that Tetrac treatment resulted in increased apoptosis levels post-ingression. Data are means±1 s.e.m. *Significance of *P*<0.05.

We conducted a two-way ANOVA with treatment and developmental stage as factors and TUNEL+ cell density as a dependent variable (Fig. 5). Both treatment (*F*_4,63_=6.554, *P*<0.001) and developmental stage (*F*_1,63_=15.217, *P*<0.001) significantly affected late-stage apoptosis, measured via TUNEL+ cell density, and the two factors had a significant interaction (*F*_4,63_=5.422, *P*<0.001). Using *post hoc* comparisons (Tukey HSD), we compared the impact of hormone treatment on late-stage apoptosis in pre- and post-ingression larvae. In pre-ingression larvae, we found that Tetrac (*X*_d_=2.762E−004; *P*=0.027) treatment resulted in higher levels of late-stage apoptosis compared with the control. In post-ingression larvae, we found that T4 (*X*_d_=−1.600E−003; *P*=0.013) treatment resulted in higher levels of late-stage apoptosis compared with the control.

To assess the difference between PO and PD arms in late-stage apoptosis of post-ingression larvae, we conducted a two-way ANOVA with treatment and arm type as factors and TUNEL+ cell density as a dependent variable. Both treatment (*F*_4,54_=6.459, *P*<0.001) and arm type (*F*_4,54_=10.791, *P*=0.002) significantly affected late-stage apoptosis, measured via TUNEL+ cell density, but no significant interaction was detected (*F*_4,54_=2.304, *P*=0.070). Late-stage apoptosis in PO (*X*_d_=−1.60E−003; *P*=0.013; see also results above) and PD (*X*_d_=−4.599E−004; *P*<0.001) arms was elevated compared with controls in response to T4 treatment. PD arms also showed increased levels of late-stage apoptosis in response to Tetrac treatment (*X*_d_=−2.666E−004; *P*=0.015). Table S1 shows all comparisons.

### T4 and Tetrac result in increased activated caspase signal in pre- and post-ingression larvae

**Fig. 6.**
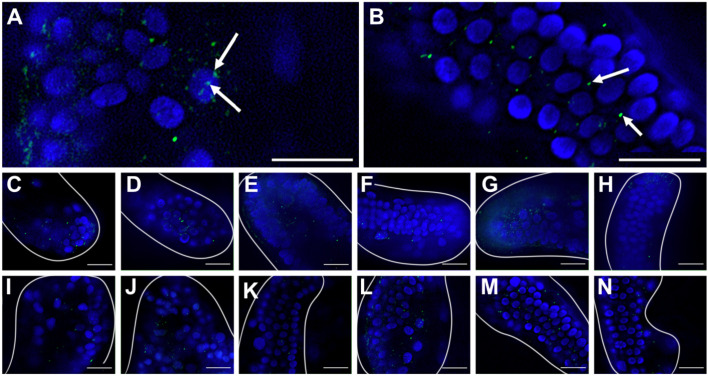
**T4 and Tetrac result in the highest number of caspase-3-positive cells compared with controls and T3 treatment.** (A,B) Representative images of caspase-3 immunohistochemistry, indicating cell-associated staining (A; arrows indicate positive staining) and staining that is not associated with cells (B; arrows indicate non-specific staining). (C–N) Representative images of caspase-3 immunohistochemistry in arms of pre-ingression (C–H) and post-ingression (I–N) larvae, exposed to control (C,I: unexposed control; D,J: positive heat shock control; E,K: rT3 control) or TH (F,L: T3; G,M: T4; H,N: Tetrac) treatment for 24 h before staining using caspase-3 antibody (green) and Hoechst (blue) and imaged at ×900 magnification. Scale bar in all images: 10 μm. Larval arms are outlined with white lines.

We measured activated caspase-3 signal in PO arms of pre- and post-ingression larvae. Fig. 6 shows representative images of PO arm staining using an activated caspase-3 antibody. As the antibody binds to the cleavage site responsible for activating caspase-3, it allows the detection of activated caspase-3 in whole-mount sea urchin embryos (Wei et al., 2012). Positive signals appeared as small green dots within cells (Fig. 6). Additionally, Hoechst was used to identify cell nuclei and therefore identify the approximate location of signals relative to the nucleus. Fluorescent signals that were near or overlapped with cell nuclei were counted as positive signals (Fig. 6A) whereas signals that were a considerable distance from any nuclei were considered to be non-specific staining (Fig. 6B). We conducted a positive control using heat shock and found that heat-shock treatment consistently resulted in elevated caspase-3 staining in larval arms (Table S1).

We conducted a two-way MANOVA to assess the impact that treatment and developmental stage have on the amount of active caspase-3 expression in the PO arms of larvae. Because the signal intensity is proportional to the amount of active caspase-3 (Wei et al., 2012), we measured both the average number of caspase-3 signals per cell (the cell-specific intensity) and the caspase-3 signal density (no. of cells µm^−1^). Both measurements of caspase-3 signal intensity increased in response to treatment (average no. of caspase-3 signals per cell: *F*_4,144_=4.175, *P*<0.001; caspase-3 signal density: *F*_4,144_=13.668, *P*<0.001) and the cell-specific intensity also increased in response to developmental stage (average no. of caspase-3 signals per cell: *F*_1,144_=17.840, *P*<0.001) but not the caspase-3 signal density (*F*_1,144_=3.283, *P*=0.072). We did not find a statistically significant interaction between treatment and stage for either signal (average no. of caspase-3 signals per cell: *F*_4,144_=0.644, *P*=0.667; caspase-3 signal density: *F*_4,144_=1.713, *P*=0.135).

**Fig. 7.**
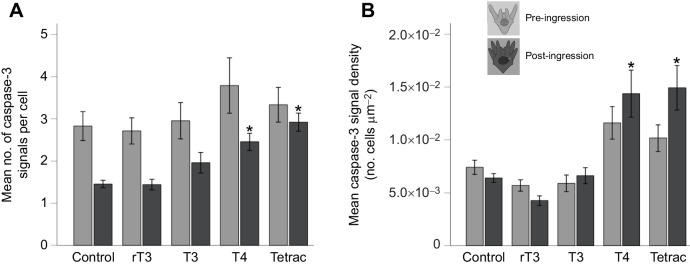
**Caspase-3 protein expression increases in response to T4 and Tetrac treatment in PO arms post-ingression but not pre-ingression.** (A) Caspase-3 immunohistochemistry measured at the per-cell level in PO arms (number of signals per caspase-positive cell) increases in T4 and Tetrac treatments compared with control and rT3 treatment. (B) Measurement of average caspase-3 signal density results a in similar pattern to that in A. Data are means±1 s.e.m. *Significance of *P*<0.05.

We further conducted *post hoc* comparisons (Tukey HSD) for pre- and post-ingression stages to compare TH treatments with the control. Pre-ingression, no treatment resulted in a significant increase of either the average number of caspase-3 signals per cell (cell-specific intensity; Fig. 7A) or the caspase-3 signal density (no. of cells µm^−1^; Fig. 7B). Post-ingression, both T4 and Tetrac resulted in an increase in the average number of caspase-3 signals per cell (*X*_d_=−1.009; *P*=0.025) and the caspase-3 signal density (*X*_d_=−1.461; *P*<0.001) compared with the control.

## DISCUSSION

The arms of larval sea urchins have critical functions in feeding and swimming (Strathmann and Grünbaum, 2006). The largest part of the ciliated band is supported by the larval arms, which are reinforced by the internal skeleton. Food availability (Boidron-Metairon, 1988; Hart and Strathmann, 1994; Strathmann, 2022), dopamine (Adams et al., 2011; Kalachev, 2020) and TH treatment (Heyland and Hodin, 2004; Heyland et al., 2006a,b) have been shown to affect the length of larval arms. Our results provide evidence that T4 and Tetrac treatment of larvae results in an increase of several forms of apoptosis in the larval arms and that this may be one mechanism contributing to the reduction of arm length in post-ingression larvae. Specifically, we showed that T4 treatment resulted in arm length reduction and increased levels of early-stage apoptosis (YP1), late-stage apoptosis (TUNEL) and caspase-3 activity in post-ingression larvae. Tetrac treatment resulted in increased levels of early apoptosis in post-ingression larvae and increased caspase-3 activity in both post- and pre-ingression larvae. We discuss these findings in the context of the developmental mechanisms underlying metamorphosis and settlement in sea urchins and other marine invertebrate species with bi-phasic life cycles and propose a mechanistic model for arm length plasticity.

### Apoptosis as a mechanism of arm length reduction in post-ingression larvae

Changes in arm length of pluteus larvae have important functional consequences for the larva with respect to growth and survival, as arm length directly impacts the amount of food that can be ingested (Boidron-Metairon, 1988; Carrier et al., 2015; Hart and Strathmann, 1994; Miner, 2005; Soars et al., 2009). Yet, arm length plasticity, i.e. the extension of arm length and the ciliated band in response to a low food environment has not been universally confirmed among echinoids (Soars et al., 2009), and the mechanisms underlying arm length reduction after extension remain unknown. Previous work on arm length plasticity has shown that echinoid species with higher levels of maternal investment show less plasticity than species with less maternal investment (Reitzel and Heyland, 2007), confirming that the dependence on exogenous food is a main driver of arm length. As pluteus larvae develop towards settlement, their dependence on exogenous food decreases and the development of the juvenile rudiment can be suspended if insufficient food is available (Carrier et al., 2015; Miner, 2005; Singh et al., 2018; Strathmann et al., 1992). Our results revealed that the effect of THs (T4 and tetrac) on arm length reduction depend on the developmental stage of the larva with little to no impact of these hormones on the arm length of pre-ingression *S. purpuratus* larvae, further emphasizing that arm length changes are intricately linked to the development of the rudiment. Previous work has also shown that THs can be synthesized by micro-algae that sea urchin pluteus larvae feed on (Chino et al., 1994; Heyland and Hodin, 2004; Heyland et al., 2006a,b; Miller and Heyland, 2013; Van Bergeijk et al., 2013). Based on our new findings, we propose that THs function as a signal, regulating and balancing investment in larval and juvenile structures of developing pluteus larvae. To test this hypothesis, additional work will be required to analyse the relationship between cell proliferation and apoptosis in larval arms and the impact of these processes on arm length in response to THs and food availability.

Echinoid larvae typically reduce their arm growth as they approach metamorphic competence and settlement (Hart and Strathmann, 1994; Heyland and Hodin, 2004, 2014; Heyland et al., 2006a, 2004; Hodin et al., 2016; Reitzel and Heyland, 2007; Soars et al., 2009; Strathmann et al., 1992). If the model outlined above is correct, this could be accomplished by reducing cell proliferation, increasing apoptosis or both. In most animals, proliferation and apoptosis are regulated by a subset of shared factors and therefore one process is linked to the other (Pucci et al., 2000). Our results provide evidence that apoptosis in larval arms increases post-ingression compared with pre-ingression and this is reflected in several measures of apoptosis, i.e. YP1, TUNEL and caspase-3 activity. Together, these findings support the idea that an increase of apoptosis post-ingression is a contributing factor to changes in arm length.

### THs differentially impact apoptosis in different cell types in larval arms

Changes in the shape and size of larval arms involve multiple tissues and cell types. Arms of pluteus larvae consist of epithelial (ciliated and non-ciliated), skeletal and mesenchymal cells. The initial larval skeleton originates from primary mesenchyme cells in the embryo and similar skeletogenic mesenchyme cells during later development (Gao and Davidson, 2008; Yajima, 2007; Yajima and Kiyomoto, 2006). These mesenchyme cells are regulated by signals from adjacent ectoderm (Sun and Ettensohn, 2014). We quantified and monitored both skeleton-associated mesenchyme cells and nearby epithelial cells for apoptosis markers in this study. Arm length changes can result from increased apoptosis in any of these cell types, yet these activities are likely coordinated between the different cell types to regulate the length of the arm. While we were able to detect a clear increase of apoptosis in ciliated and epithelial cells in response to T4 and Tetrac treatment, the response of skeletal cells was too variable to identify a clear pattern. It should be noted that Table 1 shows only the response to TH treatment in early apoptosis in the epithelial cells because they are the most reliable to measure (Table 1). This is in part due to the generally low numbers of skeletal cells scored in our experiments but could also indicate that apoptosis of the skeletal mesenchyme is not involved in the arm length changes. Photoablation of ectodermal cells at the arm tip has been shown to be sufficient to inhibit skeletal growth of the arm, as skeletogenesis is ectodermally regulated, mainly by vascular endothelial growth factor (VEGF) (Ettensohn and Malinda, 1993). It is possible that apoptosis of epithelial cells at the arm tip functions to limit skeletal growth as well as to diminish the length of the larval arm. It is not known whether skeletogenic mesenchyme cells can dissolve or remodel skeleton in larval sea urchins, but if the ectodermally produced skeletogenic signal VEGF is inhibited, the mesenchyme cells cease skeletogenesis but continue to take up calcium from their environs, accumulating calcium in vesicles (Winter et al., 2021).

Skeletal structures extending from the larval arms can often be observed when larvae have insufficient food available (H.W., E.T. and A.H., personal observation) or have been exposed to toxic compounds (Gambardella et al., 2021). In the experiments presented here, we did observe this phenotype primarily in larvae exposed to Tetrac; however, more research needs to be conducted to determine whether this indicates a lack of coordinated response between the different cell types. While treatment with T3, the active hormone for the nuclear hormone receptor in vertebrates, did not result in arm length reduction, we did detect an increase of early apoptosis in epithelial and ciliated cells of larval arms in response to T3. While the increase of early apoptosis was smaller compared with the effect of T4 and Tetrac, it was statistically significant. These findings are particularly interesting in the context of the type of signalling pathways activated by TH treatment and are discussed further below.

### T4-induced apoptosis is likely activated via non-genomic signalling

While there is no conclusive evidence for TH binding to nuclear hormone receptors in sea urchins to date, our results consistently showed stronger T4 effects on arm retraction and the expression of apoptosis markers compared with T3. In vertebrates, TH receptor proteins generally bind T3 with higher affinity than T4, but T4 has been validated as a potential integrin ligand (Davis et al., 2008; Yang et al., 2021). Additionally, the non-genomic TH response is capable of producing rapid change of the order of minutes or hours, while a genomic response to THs typically regulates gene expression over the course of days (Taylor and Heyland, 2017). Both the differential effects of THs on arm retraction and apoptosis markers, and the responses of different cell types indicate that THs may be signalling via non-genomic pathways. Previous work from our lab also indicates that T4 can bind to specific integrin subunits in the membrane and regulate skeletogenesis via phosphorylation of MAPK (ERK1/2) in PMCs (Taylor and Heyland, 2017, 2018, 2022). Therefore, our results support skeletogenesis in post-ingression larvae being at least partially regulated by non-genomic actions, rather than genomic action alone.

Similar to T4, Tetrac has been shown to bind to integrin αvβ3 (Yang et al., 2021), preventing cell proliferation and inducing apoptosis (Cohen et al., 2018). However, we were able to identify an important difference between the effects of T4 and Tetrac on apoptosis our experiments: whereas T4 treatment resulted in activation of both early (YP1) and late apoptosis markers (TUNEL) as well as caspase-3 levels, Tetrac resulted only in increased early apoptosis measured by YP1 as well as caspase-3 levels, but not late apoptosis measured by TUNEL. Exposure of human multiple myeloma cells to Tetrac typically results in caspase-9 and caspase-3 activation but not activation of the DNA-cleaving apoptosis inducing factor (AIF) (Cohen et al., 2018). Therefore, in mammals, Tetrac treatment would result in a slight decrease in DNA fragmentation and subsequent TUNEL staining, as DNA is also cleaved by a few other factors – one of the most prominent being the endonuclease DNA fragmentation factor subunit beta (DFFB) (Elmore, 2007). The lack of TUNEL activity in response to Tetrac treatment in sea urchin larvae, however, is inconsistent with data on mammals and suggests that AIF alone may be responsible for most of the DNA cleavage in sea urchins. This is verified by the fact that previous research into the apoptotic toolkit in sea urchins did not identify the presence of a DFFB orthologue (Wynen and Heyland, 2021). Future research should explore the differences and similarities of Tetrac and T4 treatment in sea urchins and also apply these hormones in combination to test whether Tetrac functions as a ligand for integrin αvβ3.

### PCD in metamorphic development as a shared evolutionary feature

The connection between THs and extensive PCD in nearly every major organ in tadpoles is probably the most well-described example of this relationship (Das et al., 2006; Ishizuya-Oka et al., 2010). This relationship has also been explored in *Drosophila* where group apoptosis regulated by ecdysone is vital for the rapid turnover of tissue (Eroglu and Derry, 2016; Fuchs and Steller, 2011). However, PCD during metamorphosis is also a common process among indirectly developing marine invertebrates as it aids in the transition from a planktonic larva to a free-living benthic juvenile (Leise et al., 2004; Gifondorwa and Leise, 2006; Hadfield, 2000; Hadfield et al., 2015; Heyland et al., 2011a; Heyland and Moroz, 2006; Lutek et al., 2018). PCD is responsible for the remodelling of the apical organ and velar lobes in the veliger larvae of indirectly developing molluscs (Heyland et al., 2011a; Kiss, 2010; Vogeler et al., 2021). In ascidians, PCD had been identified as an important part of tail regression in the transition from the larval body to the juvenile (Karaiskou et al., 2015; Weill et al., 2005). In echinoderms, PCD has been observed prior to metamorphosis in the ciliary band and larval arms (Lutek et al., 2018; Sato et al., 2006; Sutherby et al., 2012).

Hormones in general and THs specifically have broad functions in physiology and development across metazoans (Flatt et al., 2006). THs function in development, growth, differentiation, cell survival and cell death have been well documented and their role in metamorphic development of amphibians and several marine invertebrate species provides an interesting and productive framework for comparative studies, allowing the homoplasy and homology of this important signalling system to be tested (Flatt and Heyland, 2011; Flatt et al., 2006; Heyland and Moroz, 2006; Heyland et al., 2006a, 2018; Hodin, 2006; Paris et al., 2008, 2010; Wynen and Heyland, 2021; Laudet, 2011).

### Conclusion and future directions

The work presented here provides evidence that T4 is associated with an increase in PCD in the larval arms of post-ingression larvae. As T4 is associated with an acceleration of larval arm retraction prior to metamorphosis, this may provide evidence that arm retraction is mediated by apoptosis. Our work also indicates that PCD involves a compromised plasma membrane, DNA fragmentation and an increase in active caspase-3 and therefore is likely apoptosis. In combination with research in frogs and insects, our data suggest that prior to metamorphosis, TH signalling is responsible for triggering tissue remodelling through apoptosis. Our work has also shown that T3, while less effective than T4, is associated with an increase in PCD in pre-ingression larvae. Increased apoptosis in response to T4 versus T3 is consistent with previous research that suggests that T3 has a lower binding affinity in sea urchins than T4. Our research presented here has provided evidence that exposure to Tetrac may trigger apoptosis in the larval arms of post-ingression larvae but does not trigger DNA fragmentation. Our data also suggest that caspase-3 may play a non-apoptotic role in pre-ingression larvae exposed to T4; however, more work is needed to confirm this.

Future work should examine the different mechanisms of TH signalling and characterize how exposure to THs increases apoptosis. While information on the genomic mechanisms of TH signalling in sea urchins is lacking, this could be investigated by knocking out the integrin membrane receptor αvβ3 before examining the role of T4 on apoptosis in the larval arms. Examining whether T4 still triggers apoptosis in the larval arms in larvae without αvβ3 should provide evidence as to whether TH-mediated apoptosis involves genomic or non-genomic signalling. Future work should also examine whether caspase-3 plays a non-lethal role in pre-ingression larvae, similar to its role in triggering angiogenesis in certain human cells (Bernard et al., 2019). Pull-down assays should be performed to determine whether cleaved caspase-3 directly interacts with VEGF in pre-ingression sea urchin larvae exposed to T4.

Finally, while this work provides evidence that THs increase apoptosis in the larval arms prior to metamorphosis, it cannot be stated that this is the only mechanism involved in larval arm retraction. Future research should examine whether apoptosis is truly the only mechanism responsible for larval arm retraction or whether THs trigger a combination of increased apoptosis and decreased proliferation in order to facilitate larval arm retraction. This can be done by using caspase inhibitors to determine whether arm retraction still occurs without apoptosis. The role of reduced proliferation in larval arm retraction can also be measured by using DNA content quantification assays and immunohistochemistry staining for cell cycle markers. If there is no evidence of THs decreasing proliferation in post-ingression larval arms, then it may confirm the hypothesis that TH-mediated larval arm retraction is only regulated through apoptosis.

## Supplementary Material

10.1242/jexbio.244560_sup1 Supplementary information
